# Expression of Free Radicals and Reactive Oxygen Species in Endometriosis: Current Knowledge and Its Implications

**DOI:** 10.3390/antiox14070877

**Published:** 2025-07-17

**Authors:** Jeongmin Lee, Seung Geun Yeo, Jae Min Lee, Sung Soo Kim, Jin-Woo Lee, Namhyun Chung, Dong Choon Park

**Affiliations:** 1 Department of Medicine, College of Medicine, Kyung Hee University Medical Center, Seoul 02447, Republic of Korea; sallyljm@khu.ac.kr; 2 Department of Otorhinolaryngology Head & Neck Surgery, College of Medicine, Kyung Hee University Medical Center, Seoul 02447, Republic of Korea or yeo2park@gmail.com (S.G.Y.); or sunjaesa@hanmail.net (J.M.L.); 3 Department of Precision Medicine, Graduate School, Kyung Hee University, Seoul 02447, Republic of Korea; 4 Department of Convergence Medicine, College of Medicine, Kyung Hee University, Seoul 02447, Republic of Korea; 5 Medical Research Center for Bioreaction to Reactive Oxygen Species and Biomedical Science Institute, Core Research Institute, Kyung Hee University, Seoul 02447, Republic of Korea; sgskim@khu.ac.kr; 6 Department of Biochemistry and Molecular Biology, College of Medicine, Kyung Hee University, Seoul 02447, Republic of Korea; 7 Medical Science Research Institute, Kyung Hee University Medical Center, Seoul 02447, Republic of Korea; jwshsy@khmc.or.kr; 8 Department of Biotechnology, College of Life Sciences and Biotechnology, Korea University, Seoul 02447, Republic of Korea; nchung@korea.ac.kr; 9 Department of Obstetrics and Gynecology, St. Vincent’s Hospital, College of Medicine, The Catholic University of Korea, Seoul 02447, Republic of Korea

**Keywords:** reactive oxygen species, free radicals, endometriosis

## Abstract

This review explores the dual role of reactive oxygen species (ROS) and free radicals in the pathogenesis of endometriosis, aiming to deepen our understanding of these processes through a systematic literature review. To assess the induction and involvement of ROS in endometriosis, we conducted a comprehensive literature review using Cochrane Libraries, EMBASE, Google Scholar, PubMed, and SCOPUS databases. Of 30 qualifying papers ultimately reviewed, 28 reported a significant contribution of ROS to the pathogenesis of endometriosis, while two found no association. The presence of ROS in endometriosis is associated with infertility, irregular menstrual cycles, painful menstruation, and chronic pelvic discomfort. Among individual ROS types studied, hydrogen peroxide was most frequently investigated, followed by lipid peroxides and superoxide radicals. Notable polymorphisms associated with ROS in endometriosis include those for AT-rich interactive domain 1A (ARID1A) and quinone oxidoreductase 1 (NQO1) isoforms. Key enzymes for ROS scavenging and detoxification include superoxide dismutase, glutathione, and glutathione peroxidase. Effective inhibitors of ROS related to endometriosis are vitamins C and E, astaxanthin, fatty acid-binding protein 4, cerium oxide nanoparticles (nanoceria), osteopontin, sphingosine 1-phosphate, N-acetyl-L-cysteine, catalase, and a high-antioxidant diet. Elevated levels of ROS and free radicals are involved in the pathogenesis of endometriosis, suggesting that targeting these molecules could offer potential therapeutic strategies.

## 1. Introduction

Endometriosis is an estrogen-dependent disease in which glandular and stromal tissue, typically found in the endometrium, is present on parts of the body outside the uterus. Several theories have been proposed to explain its pathogenesis, including ectopic implantation of endometrial tissue, coelomic metaplasia, and induction of undifferentiated cells in the peritoneal cavity to differentiate into endometrial-like tissue. The ectopic implantation theory suggests that endometrial cells are shed during menstruation and disseminated through retrograde menstruation via the fallopian tubes, leading to their implantation at ectopic sites [1]. The coelomic metaplasia theory proposes that the coelomic epithelium undergoes transformation into endometrial tissue. This theory is supported by findings from a study on the genetic induction of endometriosis in mice, which suggested that endometriotic lesions on the ovary may arise directly from the ovarian surface epithelium through a metaplastic differentiation process induced by the activation of an oncogenic K-ras allele [2]. Finally, the induction theory suggests that endogenous biochemical factors induce undifferentiated peritoneal cells to develop into endometrial tissue, a hypothesis supported by several animal studies [3]. However, despite being a relatively common gynecological disorder, the pathophysiological mechanisms and natural course of endometriosis remain unclear. Furthermore, current diagnostic tools have insufficient sensitivity and specificity; hence, extensive research on this disease is ongoing [4].

The severity of endometriotic lesions does not necessarily correlate with the intensity of symptoms. Rather than lesion size, depth of infiltration may be more closely associated with symptom severity [5,6]. Mild endometriosis, which is characterized by mostly superficial lesions, is closely associated with cyclic local bleeding around the lesions and the action of inflammatory cytokines secreted by immune cells within the peritoneal cavity. In addition, endometriosis patients may present with cyclic symptoms in affected organs arising from hormonal influences. Several studies on patients with endometriosis have reported a strong correlation between elevated serum levels of cancer antigen 125 (CA-125), a cell surface antigen expressed by coelomic epithelium derivatives, and disease severity [7,8]. However, due to its lack of specificity for endometriosis, CA-125 has limited clinical utility in the diagnosis of this disease [9]. The most commonly used imaging modalities for diagnosing endometriosis are transvaginal ultrasonography and magnetic resonance imaging (MRI); plain X-rays and computed tomography (CT) are rarely useful in the diagnostic process. To date, the gold standard for diagnosing endometriosis has been direct visual confirmation of lesions within the peritoneal cavity via laparoscopy, with histological examination performed when necessary.

Medical treatments for endometriosis include gonadotropin-releasing hormone (GnRH) agonists, combined oral contraceptives, progestogens such as medroxyprogesterone acetate or dienogest, levonorgestrel-releasing intrauterine devices (IUDs), danazol, gestrinone, and aromatase inhibitors. Among these, no single agent has been shown to be definitively superior to others in treating pain related to surgically confirmed endometriosis. Therefore, treatment options are chosen based on individual patient factors such as side effects, compliance, and cost [10]. Although medical therapy can reduce the size of endometriomas by up to 57%, surgical treatment remains the most effective option [11]. Indications for surgical treatment include pelvic pain or dyspareunia, the expectation that ovarian endometrioma removal will increase the probability of an infertile patient becoming pregnant, and suspected tumor rupture or torsion. The principle of surgical treatment for endometriosis is the complete excision of lesions and the restoration of normal anatomical structure of the uterus, ovaries, and fallopian tubes.

## 2. Production and Role of Reactive Oxygen Species in Various Diseases

### Reactive Oxygen Species

Reactive oxygen species (ROS) are highly reactive molecules that contain oxygen, for example, hydrogen peroxide (H_2_O_2_), hydroxyl radical (OH^−^), singlet oxygen (^1^O_2_), and superoxide (O_2_·^−^). The superoxide anion is a primary ROS radical that is produced by the one-electron reduction of molecular oxygen. It is generated mainly within the mitochondria when, during the electron transport chain, electrons leak from complexes I and III and reduce oxygen prematurely. H_2_O_2_ is a non-radical ROS that is more stable than superoxide. It is formed either by dismutation of superoxide, catalyzed by the enzyme superoxide dismutase (SOD), or directly by oxidase enzymes. Unlike superoxide, hydrogen peroxide can diffuse across biological membranes, making it a significant signaling molecule. The hydroxyl radical is one of the most reactive ROS and can cause significant damage to cellular components such as DNA, proteins, and lipids. It is typically generated through the Fenton reaction, where hydrogen peroxide reacts with ferrous iron (Fe^2+^), or through the Haber–Weiss reaction involving superoxide. Due to its high reactivity, the hydroxyl radical has a very short half-life and acts primarily at its site of formation. ^1^O_2_ is an excited form of molecular oxygen with higher reactivity than the ground state. It is often produced in biological systems during photochemical reactions or by certain enzymes [12,13,14]. In the context of endometriosis, excess iron resulting from retrograde menstruation can accumulate in the peritoneal cavity, where it catalyzes Fenton reactions and leads to increased hydroxyl radical formation. Studies by Defrère and Van Langendonckt et al. have shown that iron overload in endometriotic lesions contributes to oxidative damage, peritoneal cell injury, and lesion proliferation [15].

ROS are generated as byproducts of normal cellular metabolism, particularly in the mitochondria during the ATP synthesis step of oxidative phosphorylation. Within the mitochondrial respiratory chain, complex I (NADH-ubiquinone oxidoreductase) and complex II (succinate dehydrogenase) are key sources of ROS, especially under pathological conditions. Complex I mutations can lead to excessive ROS production and disease progression, while complex II mutations contribute to ROS generation during ischemia–reperfusion injury. Complex III (ubiquinol-cytochrome c oxidoreductase) further contributes to ROS production, which can lead to apoptosis. Although mitochondria are the major source, ROS are also produced by other mechanisms, including the activation of enzymes such as NADPH oxidases (NOX), xanthine oxidoreductase (XOR), and lipoxygenases (LOXs). NOX enzymes, particularly NOX-2, generate superoxide and hydrogen peroxide during processes such as the respiratory burst in phagosomes. XOR produces ROS when its oxidase form (XO) uses oxygen as an electron acceptor, while LOXs generate ROS as byproducts during the oxygenation of fatty acids. Additionally, Toll-like receptors (TLR1, TLR2, TLR4) can enhance ROS production by recruiting mitochondria to macrophage phagosomes, and ionizing radiation (IR) generates ROS through water radiolysis, leading to the formation of free radicals [13,14,16].

To maintain homeostasis, organisms control ROS through antioxidant networks, which act as an oxidative defense system. This system includes endogenous antioxidant enzymes such as SOD, catalase (CAT), and glutathione peroxidase (GPx), which neutralize ROS to prevent inflammation. SOD is the first line of defense, converting superoxide into molecular oxygen and H_2_O_2_, which are then broken down by CAT and GPx into water and oxygen. Glutathione (GSH) is replenished via glutathione reductase (GR), supported by NADPH produced through pathways such as the pentose phosphate pathway. Although these systems efficiently eliminate O_2_^−^ and H_2_O_2_, they do not neutralize highly reactive OH^−^, making prevention of OH^−^ formation crucial for protecting organisms from oxidative damage. Low molecular mass antioxidants, such as vitamins C and E, carotenoids, and GSH, neutralize ROS directly or support ROS-detoxifying enzymes. Non-enzymatic defense mechanisms include antioxidant substances such as vitamins C and E, β-carotene, uric acid, and bilirubin. These substances react with ROS that are not targeted by enzymatic actions, such as HO· and ^1^O_2_, to neutralize their toxicity. Unlike the body’s own enzymes, such as SOD, CAT, and GPx, substances involved in non-enzymatic defense mechanisms can be supplied externally, making them important subjects of research in modern anti-aging therapies. Although ROS can contribute to diseases such as cancer and neurodegeneration when uncontrolled, at physiological levels, ROS are crucial for maintaining cellular homeostasis and regulating signaling pathways involved in growth, metabolism, and immune responses. Understanding the balance between the roles of ROS as both signaling molecules and potential agents of damage is key to developing targeted therapies that harness the dual nature of ROS [16,17,18].

**Figure 1:**
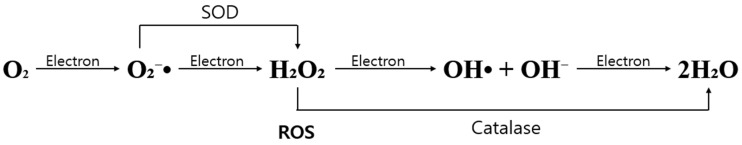
Formation of ROS and its types.

ROS are a group of free radicals formed when oxygen molecules lose one of their electrons through interactions with other molecules. When oxygen gains a single electron, it forms the highly unstable superoxide anion (O_2_^−^·). This molecule is rapidly converted to H_2_O_2_ by the action of the enzyme SOD (Figure 1).

H_2_O_2_ is more stable than superoxide, making it the predominant ROS observed in cells. Its production is usually well-regulated within the cell, with most of it detoxified into harmless H_2_O by antioxidant enzymes such as CAT and peroxidase. However, when excessive H_2_O_2_ is generated such that the cell’s regulatory capacity is insufficient to adequately remove it, H_2_O_2_ gains an additional electron to form the OH·. This highly toxic radical is the source of most of the cellular toxicity associated with ROS. Specifically, the hydroxyl radical:

- Oxidizes DNA, leading to the formation of DNA adducts
- Oxidizes lipids, generating lipid peroxides (LPOs), which contribute to cellular toxicity

In general, ROS broadly includes O_2_^−^·, H_2_O_2_, and OH· [19,20,21] (Table 1).

Therefore, various studies have examined the role of ROS in endometriosis; however, conflicting findings have been reported regarding the role and function of ROS.

While oxidative stress in endometriosis has been addressed in prior reviews, the present review provides an updated and more focused synthesis of the production patterns and mechanistic roles of ROS and free radicals. In particular, it highlights emerging molecular targets and intervention strategies based on recent studies published through late 2024, including antioxidant trials involving astaxanthin, nanoceria, and osteopontin modulation—elements not covered in earlier reviews.

## 3. Methods

Although various studies have examined the role of ROS in different diseases, a comprehensive review of the literature on the expression and role of ROS or free radicals in the development of endometriosis has not been published. Unlike previous reviews on oxidative stress in endometriosis, this narrative review focuses specifically on the expression and role of ROS and free radicals, as well as recent intervention studies targeting these molecules. Therefore, one author (J.L.) searched for studies published between January 1991 and September 2024 in five electronic databases—Cochrane Libraries, EMBASE, Google Scholar, PubMed, and SCOPUS—using the search terms ‘endometriosis’ and ‘free radicals/reactive oxygen species’. The literature search focused on studies published in English and included prospective or retrospective studies on ROS in endometriosis performed in humans or animals. The following were excluded: (1) unpublished data, (2) review articles, (3) gray literature, (4) case reports, (5) duplicates, and (6) published literature review studies by the author on the expression of nitric oxide in endometriosis [26].

**Figure 2:**
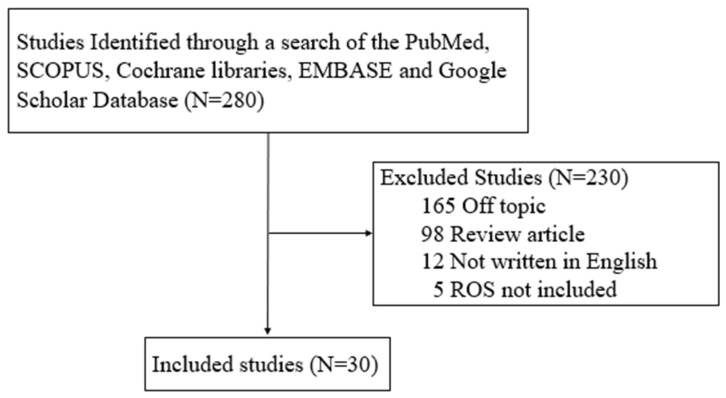
Review the flow diagram.

Our search terms/strategy identified that of the 30 studies included, 28 supported a contributory role of ROS/free radicals in endometriosis, whereas two found no significant association (Figure 2).

## 4. Role of Free Radicals and ROS in Endometriosis

To investigate the role of ROS in the pathogenesis of endometriosis, a review of relevant studies was conducted. A total of 27 papers were included in the review, and the studies were categorized based on whether ROS was reported to play a positive or no significant role in the context of endometriosis. The analysis revealed that 28 of the studies reported that ROS contributed to the pathogenesis of endometriosis, while 2 studies reported no association between ROS and the pathophysiology of the disease.

### 4.1. Studies Indicating That Increased ROS and Free Radicals Contribute to the Pathogenesis of Endometriosis

The analyzed papers were categorized and summarized into the following groups: studies related to ROS and reactive nitrogen species (RNS), studies on polymorphisms, studies involving ROS scavenging and detoxifying enzymes, and studies in which the use of inhibitors targeting ROS and free radicals led to an improvement in endometriosis.

#### 4.1.1. Studies on ROS and RNS in Endometriosis

##### Hydrogen Peroxide H_2_O_2_

One study investigated the potential regulatory effects of hydrogen peroxide (H_2_O_2_), one of the most common intracellular ROS, on endometrial cells in the context of endometriosis [27]. In the cellular environment, H_2_O_2_ acts as a signaling molecule and plays an important role in inflammatory processes. The study found that H_2_O_2_ regulates opposing cellular metabolic pathways, promoting cell proliferation at low concentrations and inducing cell death signals at high concentrations.

17β-estradiol (E_2_) is essential for cell proliferation, development, growth, and the differentiation of secondary sexual characteristics in both males and females. Estrogen is a major risk factor in the biological changes and clinical manifestations associated with endometriosis [28]; however, the biochemical mechanisms underlying the effects of estrogen on endometriosis remain unclear. Therefore, to investigate the antiapoptotic and antioxidative effects of E_2_ in endometriosis, eutopic endometrial cells were collected from 18 patients and stimulated with steady-state H_2_O_2_ ([H_2_O_2_]ss) for 20 h in the presence of E_2_. When cells were pretreated with E_2_ prior to [H_2_O_2_]ss exposure, a significant decrease in apoptosis was observed in the endometriosis group compared to the control group (*p* < 0.01), along with an increase in GSH levels. These findings suggest that, in the presence of steady-state H_2_O_2_, E_2_ exerts antiapoptotic effects in endometrial cells and may play a role in preventing the progression of endometriosis [28].

##### Hydrogen Peroxide (H_2_O_2_), Ferric-Orange Xylenol (FOX), Malondialdehyde (MDA), Glutathione (GSH), and Oxidized Glutathione (GSSG)

A cross-sectional pilot study was conducted in 33 women with endometriosis and 20 without endometriosis. Compared to eutopic endometrium, greater oxidative damage was observed in endometriotic lesions. The mean ferric-orange xylenol (FOX) value in endometriotic lesions was significantly higher than in eutopic tissue—2.15 times higher during the secretory phase (*p* = 0.001) and 2.99 times higher during the proliferative phase (*p* = 0.001). The MDA level was 2.41 times higher (*p* = 0.030) in the control group compared to eutopic tissue in the secretory phase. Additionally, both GSH and oxidized glutathione (GSSG) levels were higher in the control group. Concentration assay results showed that cell viability and proliferation significantly decreased starting from 0.25 mM, indicating that 0.25 mM was the optimal concentration for H_2_O_2_ stimulation (*p* < 0.05). Following H_2_O_2_ treatment, Lamin B1, a marker of senescence, was depleted in endometriotic lesions but was upregulated in eutopic endometrial stromal cells. These findings indicate that high levels of ROS are present in endometriotic lesions. Furthermore, stromal cells from endometriotic lesions stimulated with H_2_O_2_ exhibited stronger senescence traits compared to eutopic and non-endometriosis endometrial tissues [29].

##### Lipid Peroxides

Blood samples were collected from 40 patients aged 18 to 45 years undergoing surgery for endometriosis or tubal ligation, and the mean serum autoantibody titers to LPO-modified rabbit serum albumin, oxidized low-density lipoprotein (oxLDL), and malondialdehyde-modified low-density lipoprotein (MDA-LDL) were measured. The titer for LPO-modified rabbit serum albumin was 0.49 ± 0.12 units in the endometriosis group and 0.2 ± 0.02 units in the healthy control group. For oxidized LDL, the values were 0.22 ± 0.005 units in the endometriosis group and 0.18 ± 0.006 units in the control group. For MDA-modified LDL, the levels were 0.21 ± 0.005 units in the endometriosis group and 0.16 ± 0.003 units in the control group. These results indicate that autoantibodies to markers of oxidative stress are significantly elevated in patients with endometriosis, indicating that women with endometriosis have enhanced oxidative stress [30].

In a study of 30 infertile women undergoing diagnostic laparoscopy, 15 patients had endometriosis and 15 did not. Comparison of the LPO and SOD levels in the peritoneal fluid of these two groups showed that the LPO level in the peritoneal fluid was significantly higher in patients with endometriosis compared to the control group (*p* < 0.01), while the SOD level did not differ significantly between the two groups (*p* > 0.05). These findings suggest an imbalance between peroxidation and antioxidation in the peritoneal fluid of patients with endometriosis. They also indicate that the presence of endometriotic tissue and an increase in peritoneal macrophages may contribute to elevated LPO levels in the peritoneal fluid of patients with endometriosis. Thus, LPO is involved in the pathogenesis of endometriosis through cytotoxicity, inflammatory responses, and adhesion formation [31].

##### Oxidatively Modified Lipid–Protein Complexes

Endometrial biopsies were obtained during the follicular phase of the menstrual cycle from five women with endometriosis and five control subjects. Tissue samples were analyzed for oxidatively modified lipid proteins (HNE-7, MDA2), macrophages (HAM-56), and muscle cell actin (HHF-35). Both endometrial and endometriotic tissues showed the presence of HAM-56, MDA2, and HNE-7. While the endometrium was devoid of staining with HHF-35, some endometriotic implants exhibited patchy staining of cells with HHF-35. These findings strongly suggest the occurrence of oxidative stress in endometriotic tissues and indicate that oxidative modification in the endometrium may be part of a normal physiological process [32].

##### Production of ROS

A prospective study was conducted by collecting peritoneal fluid from women with endometriosis (*n* = 15) or idiopathic infertility (*n* = 11) who underwent laparoscopy for infertility, as well as from patients undergoing tubal ligation who served as controls (*n* = 13). ROS were detected in the peritoneal fluid of patients with endometriosis, idiopathic infertility, and tubal ligation. ROS levels in peritoneal fluid did not differ between the endometriosis and control groups, but did differ significantly between the idiopathic infertility and control groups (*p* = 0.01). These findings suggest that high levels of ROS in the peritoneal fluid may not directly cause infertility in endometriosis but may play a role in infertility in patients with idiopathic infertility [33].

##### Free Oxygen Radicals (FORT) and Free Oxidant Radical Defense (FORD)

A study was conducted on 24 women without endometriosis, 26 women with endometrioma, and 26 women with deep infiltrating endometriosis (DIE), with or without endometrioma. Levels of free oxygen radicals (FORT) and free oxidant radical defense (FORD) in capillary blood were compared among these groups. The results showed a higher oxidative stress balance (FORT/FORD ratio) in women with endometriosis compared to controls (4.75 ± 4.4 vs. 2.79 ± 2.2; *p* = 0.05), with women with DIE exhibiting the highest values (5.34 ± 4.6; *p* = 0.028 vs. controls). In the control group, hormone therapy increased both FORT levels (*p* = 0.003) and FORD levels (*p* = 0.012), but the FORT/FORD balance remained stable (2.72 ± 2.2 vs. 2.73 ± 1.8; *p* = 0.810). Among women with endometriosis, FORT levels did not differ significantly from controls, but FORD levels were significantly higher (*p* = 0.004), resulting in a lower FORT/FORD ratio (4.75 ± 4.4 vs. 2.57 ± 1.76; *p* = 0.002). These findings indicate an improvement in systemic oxidative stress balance in endometriosis. While hormonal therapy did not alter the oxidative stress balance in the control group, this balance showed improvement in women with endometriosis, particularly in patients with DIE [34].

##### O_2_^−^ + SOD + Catalase + GSH

An in vitro study was conducted using endometrial and ovarian endometrioma tissue samples from 14 patients with endometriosis, along with an animal study involving 28 eight-week-old female nude mice. Compared to controls, superoxide (O_2_^−^) production was approximately 39% higher in stromal endometriotic cells (*p* < 0.05) and about 35% higher in stromal endometrial cells (*p* < 0.05). Moreover, SOD activity was approximately three times higher in stromal endometriotic cells (*p* < 0.01) and 2.25 times higher in stromal endometrial cells (*p* < 0.05) compared to controls. In epithelial cells, SOD activity was 3.4 times higher in epithelial endometriotic cells and 2 times higher in epithelial endometrial cells compared to controls. The increase in superoxide levels in endometriotic cells was associated with a corresponding increase in SOD activity due to elevated oxidative stress.

Hydrogen peroxide levels in epithelial endometriotic cells were 7.75 times higher than in controls (*p* < 0.001) and 3.1 times higher than in epithelial endometrial cells (*p* < 0.001). Detoxification of hydrogen peroxide is mediated by GPx and CAT. CAT activity was 2.7 times lower in stromal endometriotic cells (*p* < 0.01) and 1.8 times lower in stromal endometrial cells (*p* < 0.01) than in controls. In epithelial cells, CAT activity was three times lower in epithelial endometriotic cells (*p* < 0.01) and 1.5 times lower in epithelial endometrial cells (*p* < 0.05) than in controls. Additionally, GSH levels were 1.6 times higher in stromal endometriotic cells (*p* < 0.01) and 1.2 times higher in epithelial endometriotic cells (*p* < 0.05) than in controls.

These findings indicate that endometriotic cells exhibit increased ROS production and impaired ROS detoxification, similar to tumor cells, leading to heightened oxidative stress [35].

##### Free Radical or Non-Enzymatically Derived 8-Isoprostane

A study was conducted of peritoneal fluid from 50 women aged 18–60 years who underwent either tubal ligation or laparoscopy for endometriosis. Additionally, animal and in vitro experiments were performed using eight CD-1 mice and Sprague–Dawley rats. In the peritoneal fluid of women with endometriosis, levels of free radicals or non-enzymatically derived 8-isoprostane were significantly higher compared to controls (*p* = 0.005), as were levels of 12- and 15-lipoxygenase-derived eicosanoids such as 12- and 15-hydroxyeicosatetraenoic acids (*p* < 0.05), 5-hydroperoxyeicosatetraenoic acids, prostaglandin E2, and prostaglandin D2.

In the animal study, the Hargreaves paw withdrawal assay showed a significant reduction in withdrawal latency in groups treated with prostaglandin E2, as well as in those treated with peritoneal fluid from women with endometriosis, compared to controls. In vitro experiments revealed that increased low-density lipoprotein (LDL) oxidation levels led to elevated expression of neuropathic genes involved in pain conduction—such as voltage-gated sodium channels and opioid receptor genes—as well as inflammatory genes, including interleukins IL-2 and IL-6, and the fractalkine receptor CX3CR1.

These findings suggest that non-enzymatically oxidatively modified lipoproteins play a role in thermoregulation, pain sensation, and the expression of inflammation- and pain-related genes. They also contribute to the formation of cyclooxygenase (COX)- and lipoxygenase (LOX)-derived oxidation products from polyunsaturated lipids. Based on these observations, it is proposed that antioxidants, either in combination with or as an alternative to COX inhibitors (NSAIDs), may reduce these effects [36].

Overall, studies in Section 4.1.1 consistently show that ROS—particularly hydrogen peroxide, lipid peroxides, and oxidized lipids—are elevated in endometriotic tissues and fluids, contributing to cellular damage, inflammation, and lesion progression. Many studies have highlighted an imbalance between ROS production and detoxification, demonstrating impaired catalase activity and increased oxidative stress markers. These findings support the role of ROS, not only in lesion development, but also in clinical symptoms like infertility and pain. However, a few studies report no significant ROS differences in peritoneal fluid, suggesting variability according to sample type or disease phase. This underscores the need for standardized study designs and further mechanistic research.

#### 4.1.2. Studies on Polymorphisms Related to ROS and Free Radicals in Endometriosis

An in vitro study was conducted using 31 ovarian endometriomas removed via laparoscopic surgery and 29 normal eutopic endometrium tissue samples as controls. The expression levels of genes encoding five estrogen-hydroxylating enzymes, five hydroxyestrogen-conjugating enzymes, and three estrogen quinone detoxifying enzymes were measured to investigate the oxidative metabolism of estrogen in endometriosis. Compared to normal endometrium, endometriotic tissue showed significantly increased expression of CYP1A1, CYP3A7, and COMT (*p* < 0.0001), with higher levels of MB-COMT detected. No differences were observed in the expression of CYP1B1, CYP3A5, SULT1A1, or NQO2. However, the expression levels of SULT1E1, SULT2B1, UGT2B7, NQO1, and GSPT1 were decreased in endometriotic tissue. Among the three isoforms of quinone oxidoreductase 1 (NQO1), NQO1c was identified as endometriosis-specific (*p* = 0.0007). These findings represent the first report of dysregulated expression of estrogen oxidative metabolism-related genes in ovarian endometriosis [37].

Expression of ARID1A was downregulated in the endometriosis compared to the control group. Reduced expression of the ARID1A gene was found to be associated with promoter hypermethylation. In H_2_O_2_-stimulated endometrial cells, ARID1A gene expression was decreased, indicating that ROS plays a role in regulating ARID1A expression. Additionally, both mRNA and protein levels of DNMT1 were increased in H_2_O_2_-stimulated endometrial cells. These findings suggest that, in endometriosis, downregulation of the ARID1A gene is related to promoter hypermethylation, and that this epigenetic alteration is regulated by ROS.

In another study on polymorphism, 30 endometriotic and 30 normal endometrial tissue samples were collected, and the levels of MDA, GPx, AT-rich interactive domain 1A (ARID1A), and DNA methyltransferase 1 (DNMT1) were compared. In H_2_O_2_-stimulated endometrial cells, MDA levels were decreased, whereas GPx levels were increased compared to controls [38].

The studies reviewed in Section 4.1.2 suggest that ROS are involved in epigenetic and enzymatic regulation of genes associated with estrogen metabolism and chromatin remodeling in endometriosis. Dysregulated expression of enzymes like CYP1A1, COMT, and NQO1, as well as hypermethylation-induced downregulation of ARID1A, suggest that oxidative stress alters expression patterns of genes that contribute to lesion development and hormonal imbalance. These findings support a mechanistic link between oxidative stress and the molecular alterations characteristic of endometriotic tissue.

#### 4.1.3. Studies in Which the Use of ROS Scavenging and Detoxifying Enzymes Led to a Reduction in ROS or Free Radicals, Resulting in Symptom Improvement or Therapeutic Effects in Endometriosis Patients (Table 2)

##### Mitochondrial Superoxide Dismutase 2

Endometrial tissue samples were collected from a control group of 35 women without endometriosis but with benign gynecological diseases, 78 women with endometriosis, and, in 38 of these patients, their homologous eutopic endometrium. These tissues were used to investigate the role of mitochondrial superoxide dismutase 2 (SOD2), an antioxidant enzyme located in mitochondria that plays an essential role in maintaining cellular ROS balance, beyond its antioxidant role in ovarian endometriosis.

**Table 2:** ROS scavenging and detoxifying enzymes in studies on endometriosis.

Enzyme	Reaction Catalyzed
Catalase	2H_2_O_2_ → 2H_2_O + O_2_
Cytochrome c peroxidase	complex + 2cyt-c(Fe^2+^) → enzyme + 2cyt-c(Fe^2+^) + 2OH^−^
Glutathione peroxidase	H_2_O_2_ + 2GSH → GSSG + 2H_2_O LOOH + 2GSH → GSSG +H_2_O + LOH
Glutathione S-transferase	RX + GSH → RSG + HX
Glutathione reductase	NADPH + GSSG ⇄ NADP^+^ + 2GSH
Superoxide dismutase	O_2_^•−^ + O_2_^•−^ + 2H^+^ → 2H_2_O_2_ + O_2_
Thioredoxin	Trx-(SH)_2_ + Protein-S_2_ ⇄ Trx-S_2_ + Protein-(SH)_2_

R = aliphatic, aromatic, or heterocyclic group; X = sulfate, nitrite, or halide group [19,39,40].

SOD2 expression in ectopic endometrium was significantly higher than in eutopic (*p* < 0.0001) and control (*p* < 0.0001) endometria. Additionally, in endometrial stromal cells (ESCs), SOD2 mRNA expression was elevated in ectopic ESCs compared to both eutopic ESCs (*p* < 0.01) and control ESCs (*p* < 0.01). Basal oxygen consumption rate (OCR), maximal OCR, and ATP-linked OCR were significantly reduced in si-SOD2-treated cells compared to si-Ctrl-treated cells (all *p* < 0.0001). Moreover, respiratory reserve capacity decreased following SOD2 inhibition (*p* < 0.001). These results suggest that SOD2 is crucial for maintaining mitochondrial function, as si-SOD2-treated cells exhibited impaired mitochondrial respiration and signs of damage.

In addition to the above, the number of migrated cells was significantly lower in the si-SOD2-treated group than in controls, and the migratory ability of ectopic ESCs increased following SOD2 inhibition (*p* < 0.001). Mitochondrial membrane potential (MMP), a key indicator of mitochondrial function and cellular viability, was also lower in si-SOD2-treated cells compared to the control group (*p* < 0.001).

In conclusion, mitochondrial SOD2, an antioxidant enzyme, was more highly expressed in ectopic endometrium than in normal endometrium and was shown to promote cell proliferation and migration in ovarian endometriosis. Inhibition of SOD2 expression led to decreased proliferation and migration of ectopic ESCs and increased apoptosis [41].

##### Glutathionylated Protein (GSSP), Total GSH, and Carbonic Anhydrase

Peripheral blood samples were collected from 30 women diagnosed with endometriosis through histological examination and 27 healthy volunteers as a control group. Glutathionylated protein (GSSP) content, total GSH in the cytosol, and carbonic anhydrase concentration and activity were measured. Total GSH content was decreased in the endometriosis group, whereas GSSP content was significantly increased (*p* < 0.0001). Carbonic anhydrase activity was 41 times higher in the endometriosis group than in controls (*p* < 0.0001). Additionally, the 30-kDa band was increased and the 60-kDa band was decreased in the endometriosis group (*p* < 0.0001).

These findings suggest that red blood cells (RBCs) from patients with endometriosis exhibit a higher oxidation status, and that cytosolic carbonic anhydrase may be associated with the formation of the 30-kDa monomer through oxidation-related degradation. The observed association between endometriosis and increased systemic oxidative stress and carbonic anhydrase activity could potentially serve as a diagnostic tool for assessing the oxidative status in endometriosis patients [42].

##### SOD Activity, Total Antioxidant Status, GPx Activity, and LPO

Peritoneal fluid samples were collected from 65 women, comprising 29 infertile patients with endometriosis, 12 patients with idiopathic infertility, and 24 fertile controls. SOD activity, total antioxidant status, GPx activity, and LPO levels were measured. Among the groups, infertile patients with endometriosis showed the lowest levels of SOD, GPx, and total antioxidant status, while exhibiting the highest LPO levels. In contrast, patients with idiopathic infertility had the highest levels of SOD, GPx, and total antioxidant status, and the lowest LPO levels.

These findings suggest that the low total antioxidant status and decreased activity of antioxidant enzymes in the peritoneal fluid of infertile women with endometriosis may contribute to the development and progression of endometriosis [43].

The studies in Section 4.1.3 highlight the crucial role of antioxidant enzymes such as SOD2, GPx, GSH, and carbonic anhydrase in regulating oxidative stress and maintaining cellular homeostasis in endometriosis. Increased SOD2 expression in ectopic lesions supports a role for this enzyme in mitochondrial function and cell proliferation, whereas systemic markers like elevated glutathionylated proteins and carbonic anhydrase activity indicate widespread oxidative imbalance in affected individuals. Notably, infertile women with endometriosis exhibit reduced peritoneal antioxidant capacity and elevated lipid peroxides, suggesting a pathogenic link between impaired detoxification and disease progression. These findings suggest that enhancing antioxidant defenses may hold therapeutic value in endometriosis management.

#### 4.1.4. Studies in Which the Use of Inhibitors Targeting ROS and Free Radicals Led to an Improvement in Endometriosis (Table 3)

##### Macrophages

An in vitro study was conducted using cyst walls of ovarian endometriomas collected from 14 women during surgery for endometriosis. Endometriotic cells were cultured with or without differentiated macrophages (dHTP-1 cells) and treated with hydrogen peroxide or methemoglobin, two major components of endometriotic cyst fluid. Endometriotic cells co-cultured with dHTP-1 cells exhibited differentially expressed genes (DEGs) compared to monocultures. In the co-cultured endometriotic cells, TGF-β1 protein was found to be upregulated. TGF-β1, in turn, promoted the expression of heme oxygenase-1 (HO-1) in dHTP-1 cells.

Compared to dHTP-1 monoculture, HO-1 expression was significantly increased in dHTP-1 cells co-cultured with endometriotic cells. Treatment with H_2_O_2_ or methemoglobin led to elevated HO-1 protein levels in dHTP-1 cells, with even greater upregulation observed when dHTP-1 cells were co-cultured with endometriotic cells. Co-culture with dHTP-1 cells provided protective effects for endometriotic cells against oxidative injury. When HO-1 was inhibited, the protective effect of macrophages was lost.

**Table 3:** ROS inhibitors and ROS promoters used in endometriosis research.

Regulator Type	Agents	Effects
ROS inhibitors	ERK HIF-2α NRROS Nrf2 PKM2 PGC-1α Ucp2 Vitamin C/E, FHC	Reduce oxidative damage to cardiac cells ROS homeostasis Reduce tissue damage Limit ROS production in tumors Reduce oxidative damage in lung cancer cells Activate antioxidant enzymes Limit inflammation and ROS production in macrophages Suppress ROS accumulation
ROS promotors	EST-1 MMP-3 NOX2 P66^SHC^ TNF TLR 1,2,4 UPBEAT1	Increase ROS generation DNA damage and genomic instability Increase mitochondrial ROS production Increase mitochondrial ROS as an apoptosis signal Enhance macrophage killing and necroptosis Increase ROS generation in macrophages Change cells from proliferation to differentiation

Abbreviations: ERK, Extracellular signal-Regulated Kinase; EST-1, ETS-domain transcription factor 1; FHC, Ferritin Heavy Chain; HIF-2α, Hypoxia-Inducible Factor 2 Alpha; MMP-3, Matrix Metalloproteinase-3; NOX2, NADPH Oxidase 2; NRROS, Negative Regulator of Reactive Oxygen Species; Nrf2, Nuclear factor erythroid 2–related factor 2; P66^SHC^, 66-kDa Isoform of the SHC-transforming Protein; PCG-1α, Peroxisome Proliferator-Activated Receptor Gamma Coactivator 1-Alpha; PKM2, Pyruvate Kinase M2 Isoform; TLR, Toll-Like Receptor; TNF, Tumor Necrosis Factor; Ucp2, Uncoupling protein 2; UPBEAT1, Upstream Binding Transcription Factor 1.

These results demonstrate that TGF-β1 produced by endometriotic cells protects them from oxidative injury by upregulating macrophage-derived HO-1. This finding suggests that macrophage-derived HO-1 could be a potential therapeutic target in endometriosis [44].

##### Vitamins

To investigate the effect of vitamin C on oxidative stress markers in serum and follicular fluid (FF), 280 patients with endometriosis undergoing in vitro fertilization–embryo transfer (IVF-ET) were randomly divided into a vitamin C treatment group (*n* = 160) and a non-treatment group (*n* = 120). An additional 150 patients without endometriosis who underwent IVF-ET served as the control group.

Levels of vitamin C, SOD, and total antioxidant capacity (TAC) in FF were significantly lower in patients with EM compared to the control group (*p* < 0.05). Although serum levels of vitamin C, SOD, and TAC were slightly lower in the EM group than in controls, the differences were not statistically significant. Levels of SOD and TAC in FF were significantly higher than the corresponding serum levels (*p* < 0.05). Conversely, serum MDA levels were higher than FF MDA levels in both groups (*p* < 0.05), and FF MDA levels were significantly higher in the EM group than in controls (*p* < 0.05). ROS levels in both serum and FF were also significantly elevated in the endometriosis group (*p* < 0.05) [45].

In summary, in patients with endometriosis, FF levels of ROS and MDA were significantly increased, while vitamin C, TAC, and SOD levels were significantly decreased compared to normal controls. The observation of higher levels of SOD and TAC in FF compared to serum, along with lower MDA levels in FF than in serum, suggests that FF may have a stronger antioxidant capacity to protect oocytes from oxidative stress. These findings indicate that increasing FF vitamin C levels through vitamin C supplementation could potentially improve the quality of oocytes and embryos.

Four studies have investigated the effects of vitamins, specifically vitamins C and E, in endometriosis. In the first study, a prospective clinical cohort study was conducted using blood samples collected from 23 infertile women with endometriosis and 68 controls, including infertile women without endometriosis. Vitamin C levels in the FF were significantly lower in the endometriosis group compared to the control group (12.7 ± 5.9 vs. 9.7 ± 6.9 mg/mL, *p* = 0.003), and there was a trend toward reduced plasma SOD activity (0.9 ± 1.4 vs. 0.5 ± 0.7 U/mL, *p* = 0.059). Interestingly, plasma vitamin E levels were significantly higher in the endometriosis group (8.1 ± 3.8 vs. 5.2 ± 3.2 mg/mL, *p* = 0.001). These findings suggest that infertile women with endometriosis have reduced antioxidant capacity, and that an imbalance between pro-oxidants and antioxidants may negatively impact folliculogenesis and embryonic development, contributing to lower-quality oocytes and embryos in women with endometriosis [46]. In the second study, 59 women aged 19–41 years were randomly divided into two groups. Group A (*n* = 46) received a combination of vitamin E (1200 IU) and vitamin C (1000 mg), while group B (*n* = 13) received placebo pills. After antioxidant treatment, 43% of patients in the treatment group experienced improvement in chronic pain (*p* = 0.0055), and 37% and 24% of patients showed improvements in dysmenorrhea and dyspareunia, respectively. In the placebo group, by contrast, only 4 of the 13 women experienced improvement in dysmenorrhea-related pain, with no woman reporting a change in chronic pain or dyspareunia. Additionally, the antioxidant-treated group showed significantly reduced levels of peritoneal fluid markers, including RANTES (*p* ≤ 0.002), interleukin-6 (*p* ≤ 0.056), and monocyte chemotactic protein-1 (*p* ≤ 0.016), indicating that antioxidants such as vitamins C and E may reduce oxidative stress and alleviate chronic pelvic pain in endometriosis patients [47]. The third study was a randomized controlled trial involving 34 women with endometriosis. Participants were randomly assigned to either a vitamin supplementation group receiving vitamins C (343 mg) and E (84 mg) (*n* = 16) or a placebo group (*n* = 18). Plasma and peritoneal fluid were collected for analysis. After 4 months, the treatment group showed significantly lower levels of MDA and lipid hydroperoxides (LOOHs) compared to the control group (*p* < 0.05). MDA, an indicator of overall lipid peroxidation, showed a significant decrease compared to baseline after 4 months of vitamin C + E treatment, whereas LOOH, a marker of oxidative stress in peripheral compartments, showed a significant reduction after 6 months. These findings suggest that the observed reductions in MDA and LOOH levels were due to the antioxidant effects of vitamins C and E. The study concluded that vitamin supplementation can influence both the peritoneal environment and the peripheral compartment, effectively reducing oxidative stress [48]. In the fourth study, 60 reproductive-aged women (15–45 years old) with pelvic pain were randomly assigned to two groups. Group A (*n* = 30) received vitamin C (1000 mg/day, two 500 mg tablets) and vitamin E (800 IU/day, two 400 IU tablets), while group B (*n* = 30) received placebo pills. MDA and ROS levels were measured in serum and FF. Compared to the control group, Group A showed significantly lower MDA (*p* = 0.002) and ROS (*p* < 0.001) levels, although there was no change in TAC.

Post-treatment, visual analog scale (VAS) scores for dysmenorrhea (*p* < 0.001), dyspareunia (*p* < 0.001), and chronic pelvic pain (*p* < 0.001) were significantly lower in Group A than in the control group. Group B also showed reductions in dysmenorrhea (*p* < 0.001) and dyspareunia (*p* < 0.001), but chronic pelvic pain slightly increased (*p* = 0.571). Of the two groups, Group A showed a greater reduction in VAS scores for dysmenorrhea, dyspareunia, and chronic pelvic pain.

The above results suggest that vitamin C and E supplementation can reduce systemic oxidative stress in women with endometriosis and may be a useful adjunct in managing the condition. The findings suggest that antioxidant supplementation has the potential to improve the oxidative stress state and alleviate chronic pelvic pain, dysmenorrhea, and dyspareunia in endometriosis patients [49].

##### Genistein

To explore the effects of genistein on endometriosis in mice, 32 healthy female mice (Mus musculus) were divided into a negative control group, an endometriosis group, and a treatment group receiving different doses of genistein. The experimental procedures included quantitative colorimetric determination and colorimetric assays to measure the levels of SOD and GPx.

The results showed that endometriosis model mice exhibited significantly lower levels of SOD (*p* = 0.006) and GPx (*p* = 0.023) compared to the control group. Administration of genistein normalized these differences, with significantly increased SOD levels observed in the treatment group at doses of 0.13 mg (*p* < 0.001) and 0.26 mg (*p* < 0.001). GPx levels were also significantly increased across all treatment groups. The observation of reduced enzyme levels in the peritoneal fluid of the endometriosis group indicates decreased antioxidant capacity in the endometriosis model. Administration of genistein increased SOD and GPx levels in the peritoneal cavity, and its scavenging ability may help alleviate oxidative stress. Therefore, genistein may be beneficial in the treatment of endometriosis [50]

##### Astaxanthin

To evaluate the effects of astaxanthin (AST) therapy, a randomized controlled trial was conducted involving 50 infertile endometriosis patients undergoing ART. Blood serum and FF samples were collected. The results showed that, after 12 weeks of AST treatment, TAC levels (398.661 ± 57.686 vs. 364.746 ± 51.569; *p* = 0.004) and SOD levels (13.458 ± 7.276 vs. 9.040 ± 5.155; *p* = 0.010) had increased significantly. Serum MDA levels decreased following antioxidant treatment (14.619 ± 2.505 vs. 15.939 ± 1.512; *p* = 0.031). Additionally, serum levels of inflammatory markers such as IL-1β (*p* = 0.000), IL-6 (*p* = 0.024), and TNF-α (*p* = 0.038) were significantly reduced after AST therapy. AST supplementation also led to an increase in the numbers of oocytes retrieved (10.48 ± 6.665 vs. 6.72 ± 4.3; 14.60 ± 7.79 vs. 9.84 ± 6.44; *p* = 0.043) and mature (MII) oocytes (4.52 ± 2.41 vs. 2.72 ± 2.40; *p* = 0.024).

These findings suggest that AST treatment can modulate inflammation and oxidative stress in infertile patients with endometriosis, and that AST may serve as a potential therapeutic target for patients with endometriosis undergoing ART [51].

##### miR-455 Targets FABP4

An in vitro study using immortalized human endometrial stromal cells (HESCs) found that when HESCs were treated with hydrogen peroxide (H_2_O_2_), miR-455 levels decreased in a dose-dependent manner. Upregulation of miR-455 significantly reduced H_2_O_2_-induced apoptosis in HESCs, and the H_2_O_2_-induced cleavage of caspase-3 was attenuated by miR-455 overexpression. These findings indicate that miR-455 plays a protective role against H_2_O_2_-induced apoptosis in HESCs. Furthermore, miR-455 reduced the H_2_O_2_-induced increase in intracellular MDA levels. It also inhibited the H_2_O_2_-induced decrease in the activity of endogenous antioxidative enzymes such as SOD, CAT, and GPx, suggesting that miR-455 can alleviate oxidative stress in HESCs. Overexpression of miR-455 significantly decreased both the mRNA and protein levels of fatty acid-binding protein 4 (FABP4) in HESCs. Conversely, overexpression of FABP4 diminished the protective effects of miR-455 against H_2_O_2_. Additionally, forced expression of miR-455 reduced levels of ROS and MDA, while promoting the activities of SOD, CAT, and GPx.

In conclusion, miR-455 was shown to protect HESCs from oxidative stress, at least in part, through the regulation of FABP4 [52].

##### Cerium Oxide Nanoparticles (Nanoceria)

The effects of cerium oxide nanoparticles (nanoceria) on endometriosis were investigated in 60 CD-1 strain Swiss Albino mice divided into control and endometriosis-induced groups. Blood and peritoneal tissue samples were collected. Nanoceria are known for their unique ability to scavenge free radicals—particularly superoxide radicals and hydrogen peroxide—both in vitro and in vivo. In endometriosis-induced mice, levels of ROS (*p* < 0.05) and LPO (*p* < 0.001) were significantly increased compared to controls, while TAC levels were significantly decreased (*p* < 0.001). Angiogenesis markers such as adrenomedullin (ADM) and vascular endothelial growth factor (VEGF) were also elevated (*p* < 0.001). In endometriotic mice, treatment with nanoceria significantly decreased ROS levels (*p* < 0.05), but N-acetyl cysteine (NAC) injection did not. Nanoceria treatment also reduced LPO levels and increased TAC levels (*p* < 0.05). Moreover, nanoceria resulted in a nearly three-fold decrease in ADM and a significant reduction in VEGF. These findings suggest that nanoceria can mitigate endometriosis-related oxidative stress and its adverse effects on oocyte quality, supporting the potential application of these nanoparticles in the treatment of endometriosis [53].

##### Osteopontin

An in vivo study was conducted using endometrial stromal cells collected from 20 patients diagnosed with endometriosis and 10 control subjects without endometriosis. Additionally, an in vitro study was performed using endometriotic stromal cells isolated from endometrial samples and menstrual blood-derived endometrial cells (MESCs). Compared to the control group, significant upregulation of osteopontin (OPN) was observed in the endometriosis group (*p* < 0.0001). Knockdown of OPN led to the suppression of proinflammatory factors such as IL-1β, TNF-α, IL-6, IL-8, and IL-15, whereas anti-inflammatory factors such as IL-10 were upregulated. These results indicate that OPN promotes the proliferation, migration, and invasion of inflammatory factors in endometriosis ectopic stromal cells (EESCs).

Furthermore, OPN knockdown reduced the levels of necroptosis marker proteins and lactate dehydrogenase (LDH) in EESCs, suggesting that OPN promotes necroptosis. However, OPN knockdown alone also induced cell death, indicating that necroptosis is not the sole mechanism of OPN-mediated cell death. The study demonstrated that OPN knockdown suppressed mitochondrial stress and ROS release, thereby inhibiting the secretion of inflammatory factors. This process was mediated through RhoA signaling. RhoA was found to be involved in OPN-regulated mitochondrial stress and played a role in alleviating oxidative stress by suppressing the mitochondrial death pathway. In summary, OPN knockdown inhibited necroptosis through the Rho/ROS signaling pathway and suppressed endometriosis progression, necroptosis, ROS production, and inflammatory factor release. These findings suggest that OPN regulates the RhoA/ROS signaling pathway, controlling both necroptosis and the release of inflammatory mediators, and that targeting OPN may offer a potential therapeutic strategy for endometriosis [54].

##### Sphingosine 1-Phosphate

An in vitro study was conducted using immortalized HESCs. Treatment with 100 nM sphingosine-1-phosphate (S1P) for 10 min significantly increased ROS formation in HESCs, with an approximately 40% increase in ROS levels (*p* < 0.001). This brief stimulation also activated the MEK5, SRC, and ERK1/2 pathways in HESCs, and it was found that S1P activates ERK5 through engagement of S1P1 and S1P3 receptors (*p* < 0.05). Activation of ERK5 via its active upstream kinase MEK5 (MEKDD) further enhanced intracellular ROS levels, indicating that MEK5-dependent phosphorylation of ERK5 modulates ROS levels in HESCs. This S1P-induced intracellular ROS production was inhibited by siRNA targeting S1P1 and S1P3, as well as by pharmacological blockade of these receptors (*p* < 0.001). Additionally, inhibition of ERK5 reduced the S1P-dependent increase in the proinflammatory cytokines IL-1β and IL-6. Similar reductions were observed when cells were pretreated with the ROS scavenger N-acetylcysteine (NAC), suggesting that ROS plays a mediating role in this inflammatory response. In conclusion, S1P signaling via ERK5 activation contributes to a proinflammatory response in the endometrium and highlights the S1P/ERK5 pathway as a potential therapeutic target in endometriosis [55].

##### CAT and NAC

Fifty Wistar rats were divided into four groups: an endometriosis (EMs) group, an EMs group treated with normal saline, an EMs group treated with N-acetylcysteine (NAC), and an EMs group treated with CAT. Endometriotic endometrial tissue was collected, and levels of NAC and CAT were analyzed.

In the EMs group, LC3 levels were significantly reduced compared to the control group (*p* < 0.05). However, both the NAC and CAT treatment groups showed downregulation of Beclin-1 protein (*p* < 0.05) and significantly lower levels of ROS (*p* < 0.05). These findings indicate that hypoxia-induced autophagy in EM cells and ROS generation can be reversed by treatment with CAT and/or NAC. The study thus suggests that antioxidant therapy and autophagy regulation may be used to prevent the progression of endometriosis [56].

##### Peripheral Antioxidant Markers

A comparative cross-sectional study was conducted on blood samples collected from women with endometriosis (WEN, *n* = 83) and without endometriosis (WWE, *n* = 80). The WEN group was further divided into a control (*n* = 35) and a high antioxidant diet (HAD) group (*n* = 37).

When comparing WEN and WWE, the WEN group had significantly lower intake of vitamins A, C, and E, as well as zinc and copper (*p* < 0.05). Among all groups, the HAD group had the highest intake of the three vitamins. Compared to the control, the HAD group showed higher concentrations of vitamins (including serum retinol, alpha-tocopherol, and leukocyte and plasma ascorbate), enhanced antioxidant enzyme activity (SOD and GPx), and decreased levels of oxidative stress markers (MDA and lipid hydroperoxides). These findings suggest that consumption of a high antioxidant diet may improve oxidative stress status in women with endometriosis [57] (Table 4).

Collectively, studies in Section 4.1.4 demonstrate that a wide range of ROS-targeting interventions—including vitamins C and E, astaxanthin, genistein, nanoceria, and biological regulators like miR-455 and osteopontin—can reduce oxidative stress and alleviate key clinical symptoms of endometriosis. These interventions were shown to lower ROS and MDA levels, enhance the activity of antioxidant enzymes (e.g., SOD, GPx), and improve oocyte quality and pain scores in both human and animal models. Molecular targets such as HO-1, FABP4, and the S1P-ERK5 signaling axis have also been implicated in modulating ROS-mediated inflammation. Notably, some strategies (e.g., miR-455 overexpression, OPN knockdown, nanoceria therapy) simultaneously target both oxidative and inflammatory pathways, supporting their potential as multi-modal therapeutic approaches. These findings support the growing rationale for antioxidant-based and targeted redox-modulating therapies in managing endometriosis.

### 4.2. Studies Suggesting That ROS Are Not Associated with the Pathogenesis of Endometriosis

Twelve peritoneal fluid samples from patients with moderate to severe endometriosis, 15 samples from patients with minimal to mild endometriosis, and 13 from patients without endometriosis were analyzed for LPO (MDA) levels. LPO levels were not affected by the presence or severity of endometriosis, suggesting that LPOs in the pelvic cavity may not play a significant role in the association between endometriosis and infertility [58].

In another study, peritoneal fluid samples were collected from 21 infertile women with endometriosis and 21 patients without endometriosis undergoing tubal ligation. Lipid peroxidation levels, including MDA, MDA with copper addition, and cholest-3,5-dien-7-one, were measured. No significant differences in lipid peroxidation levels were observed according to the stage of endometriosis (*p* > 0.05), and no significant differences were found between patients with endometriosis-related infertility (0.07 nmol/mL, 0.34 nmol/mL, 0.24 mg/mL, respectively) and disease-free controls (0.04 nmol/mL, 0.21 nmol/mL, 0.25 mg/mL, respectively).

These results indicate that LPO levels do not differ between women with endometriosis-related infertility and fertile disease-free controls, suggesting that increased ROS in endometriosis may not be directly related to impaired fertility in these patients [59] (Table 5).

## 5. Summary

In total, 30 studies were reviewed to investigate the role of ROS in the pathogenesis of endometriosis. Among these, 28 studies found that ROS contributed to the pathogenesis of endometriosis, whereas 2 studies concluded that ROS were not associated with the pathophysiology of the disease. The key findings of the 30 studies can be summarized as follows:

(1) The primary ROS involved in the pathogenesis of endometriosis is hydrogen peroxide (H_2_O_2_), LPOs, and superoxide radicals.
(2) Polymorphism-related studies in endometriosis associated with oxidative stress included genes such as AT-rich interactive domain 1A (ARID1A) and quinone oxidoreductase 1 (NQO1) isoforms.
(3) In studies showing symptom improvement or therapeutic effects in endometriosis patients through the reduction in ROS and/or free radicals, the ROS scavenging and detoxifying enzymes used included SOD, GSH, and GPx.
(4) In studies where inhibitors of ROS and free radicals led to improvement in endometriosis, the agents used included vitamins C and E, astaxanthin, FABP4, cerium oxide nanoparticles (nanoceria), osteopontin, S1P, NAC, CAT, and high antioxidant diets.
(5) In the studies that found no association between ROS and the pathogenesis of endometriosis, the substances examined were lipid peroxidation markers, including MDA, MDA with copper addition, and cholest-3,5-dien-7-one. These markers showed no variations according to the stage of endometriosis and were not associated with reduced fertility in patients with endometriosis.

**Figure 3:**
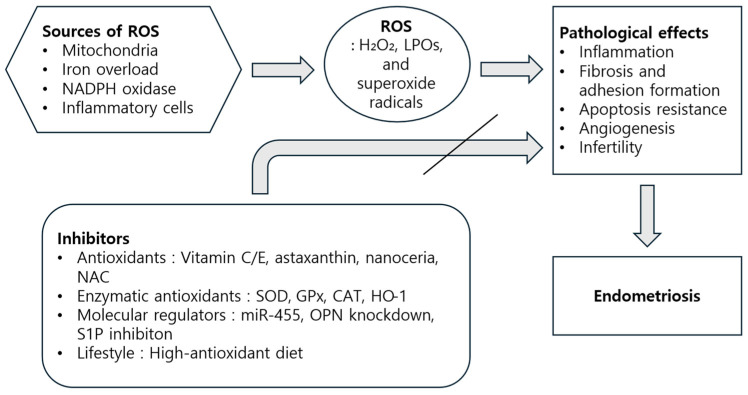
Schematic overview of the role of reactive oxygen species (ROS) in the pathogenesis of endometriosis and the potential sites of antioxidant intervention.

Based on the key concepts discussed throughout this review, we constructed Figure 1 to illustrate the mechanistic pathway of ROS in endometriosis and potential points of therapeutic intervention (Figure 3).

## 6. Conclusions

While a few studies suggest that ROS or free radicals may not be directly involved in the pathogenesis of endometriosis, the preponderance of research indicates a significant association between ROS and the development of the disease. This association extends to clinical manifestations such as infertility, irregular menstrual cycles, and pelvic pain. Our review synthesizes findings that highlight the critical role of ROS in these processes. Moreover, recent research into antioxidant therapies suggests promising avenues for regulating ROS and free radicals, potentially leading to interventions that prevent the onset and progression of endometriosis. These insights could lead to improved management of the disease and its symptoms, offering new hope for affected individuals. Future studies should continue to explore these therapeutic strategies to validate and expand upon these findings.

## Figures and Tables

**Table 1:** Reactive oxygen species and reactive nitrogen species in studies on endometriosis.

	Reactive Oxygen Species		Reactive Nitrogen Species	
Free Radicals	O_2_^−^ HO_2_^−^ H_2_O_2_ HO^−^ ^1^O_2_ OCl^−^ O_3_	Superoxide radical (or anion) Perhydroxyl radical Hydrogen peroxide Hydroxyl radical Singlet oxygen Hypochlorite Ozone	NO NO_2_	Nitric oxide Nitrogen dioxide
Non-Free Radicals	H_2_O_2_ HOCl ^1^O_2_	Hydrogen peroxide Hypochlorous acid Singlet oxygen	ONOO NO_2_^−^ NO_3_^−^	Peroxynitrite Nitrite Nitrate

Table [22,23,24,25].

**Table 4:** Research studies suggest that an increase in ROS is involved in the pathogenesis of endometriosis.

Author/ Year/ Reference	Study Design	Species and/or Sample	Detection Method	Target Substances	Results/Conclusions
Andrade et al., 2013 [26]	In vitro	Tissue samples were collected from 7 patients without endometriosis as controls and 11 patients with endometriosis who were reported to suffer from chronic pelvic pain.	Tissue isolation and cell culture, ferric-xylenol orange assay, ultraviolet spectroscopy, lactate dehydrogenase assay, Western blot analysis	H_2_O_2_, catalase	When endometrial cells were pretreated with E_2_ and [H_2_O_2_]ss, a decrease in apoptosis was observed compared to control cells (*p* < 0.01). Endometrial cells from patients with endometriosis subjected to both E2 and [H_2_O_2_]ss showed increased ERK phosphorylation. /These findings suggest that H_2_O_2_ is a signaling molecule that downregulates apoptosis in endometrial cells, supporting the fact that endometriosis, although a benign disease, shares features with cancer, such as decreased catalase levels. These results link the effects of E_2_ on [H_2_O_2_]ss to resistance to apoptosis and progression of endometriosis.
Shanti et al., 1999 [29]	Prospective study	Blood samples were collected from 40 patients aged 18–45 years undergoing laparoscopy or laparotomy.	ELISA	LPO, MDA, oxidized LDL	Mean serum autoantibody titers to the three antigens were as follows: (1) LPO-modified rabbit serum albumin, 0.49 ± 0.12 units in endometriosis patients and 0.2 ± 0.02 units in the controls; (2) oxidized low-density lipoprotein, 0.22 ± 0.005 units in endometriosis patients and 0.18 ± 0.006 units in controls; and (3) malondialdehyde-modified low-density protein, 0.21 ± 0.005 units in endometriosis patients and 0.16 ± 0.003 units in controls. /Autoantibodies to markers of oxidative stress were significantly increased in women with endometriosis. These findings strongly suggest that women with endometriosis have enhanced oxidative stress.
Yi et al., 2001 [30]	Comparative study	Peritoneal fluid was collected from 30 infertile women undergoing diagnostic laparoscopy, including 15 patients with endometriosis and 15 control subjects.	ELISA	Lipid peroxide, superoxide dismutase	The level of LPO in peritoneal fluid from patients with endometriosis was significantly higher than that of infertile women with normal pelvises (*p* < 0.01). The SOD level of peritoneal fluid showed no significant difference between the two groups (*p* > 0.05). /LPO is abundant and participates in the pathogenesis of endometriosis through cytotoxicity, inflammatory response, and adhesion formation.
Murphy et al., 1998 [31]	Controlled clinical study	Five women with endometriosis had laparoscopic resection of endometriosis and underwent biopsy. Five controls underwent endometrial biopsy in the follicular phase of the cycle (endometrial tissue sample).	Immunocytochemistry	Oxidatively modified lipid proteins (HNE-7, MDA2), macrophage (HAM-56), muscle cell actin (HHF-35)	Both endometrium and endometriosis tissues contained stromal cells that immunostained with HAM-56 and showed immunostaining (both intracellular and extracellular) with HNE-7 and MDA2. Some endometriotic implants showed patchy staining with HHF-35. The endometrium was devoid of staining with HHF-35. When stained with nonimmune sera as a control, both tissues were devoid of staining. /These data strongly indicate the presence of oxidative stress in endometriotic tissue. Ectopic endometrium may be a possible source of oxidized lipid proteins in the peritoneal cavity by diffusion or as a result of induction by tissue macrophages.
Malvezzi et al., 2023 [32]	Cross-sectional pilot study	Endometrial tissue was collected from 53 patients who were divided into two groups: 33 patients with endometriosis and 20 patients without endometriosis.	FOX assay, protein quantitation, ELISA, IHC, GSH and GSSG absorption assay, stromal cell culture, immunofluorescence, β-galactosidase assay	MDA, H_2_O_2_	Higher oxidative damage was observed in endometriotic lesions than in eutopic endometrium. Concentration assays suggested 0.25 mM to be the ideal H_2_O_2_ stimulus, as there was a significant drop (*p* < 0.05) in cell viability and proliferation when going to higher concentrations. H_2_O_2_-treated stromal cells increased the relative expression of p16^ink4a^. /These results indicate the presence of higher ROS levels in endometriotic lesions and the upregulation of MAPK. In addition, endometriotic lesions in stromal cells stimulated with hydrogen peroxide developed more senescence traits than eutopic and non-endometriosis endometrium.
Wang et al., 1997 [33]	Prospective study	Peritoneal fluid was collected from women with endometriosis (*n* = 15) or idiopathic infertility (*n* = 11) who underwent laparoscopy for infertility, and from patients undergoing tubal ligation who served as controls (*n* = 13).	Peroxidase staining test, chemiluminescence	PMN granulocyte, macrophage, ROS	ROS were present in the peritoneal fluid of patients with endometriosis, idiopathic infertility, and tubal ligation. ROS levels did not differ significantly between patients with endometriosis and the control group in unprocessed peritoneal fluid, but did differ significantly between patients with idiopathic infertility and controls in processed peritoneal fluid. /A lack of antioxidant enzymes or low antioxidant capacity may be responsible for the observed increase in ROS levels.
Biasioli et al., 2022 [34]	Prospective cohort study	Capillary blood samples were collected from 24 women without endometriosis, 26 women with endometrioma, and 26 women with deep infiltrating endometriosis (DIE), with or without endometrioma.	Colorimetric analysis, spectrophotometer,	Free oxygen radicals (FORT), free oxidant radical defense (FORD)	Women were prescribed contraceptive hormones, and the baseline assessments were repeated at the third month of use, revealing a higher oxidative stress balance (FORT/FORD) in women with endometriosis than in controls (*p* = 0.05). Regression analysis revealed an independent link between FORT/FORD and endometrioma (*p* = 0.027) and DIE (*p* = 0.001) but a negative correlation with HDL-cholesterol (*p* = 0.043). In women with endometriosis, FORT remained unchanged, but FORD increased (*p* = 0.004), and the FORT/FORD ratio significantly decreased to values similar to the control levels (*p* = 0.002). /The results indicate that women with endometriosis, particularly those with DIE, have increased systemic oxidative stress, as assessed by the ratio of free oxygen radicals (FORT) to antioxidants (FORD). The greater oxidative stress seen in these women is improved by the administration of estradiol-based hormonal contraceptives.
Ngo et al., 2009 [35]	In vitro, animal study	Endometrium and ovarian endometrioma specimens were obtained from 14 patients with endometriosis undergoing surgical treatment. Twenty-eight 8-week-old female nude mice were studied.	Cell culture, ultraviolet spectroscopy, in vitro cell proliferation and viability assay, immunoblotting	Superoxide anion, hydrogen peroxide, SOD, GSH, CAT	The production of O_2_^−^ was increased by 39% in stromal endometriotic cells from patients (*p* < 0.05) and by 35% in stromal endometrial cells (*p* < 0.05) compared with stromal control cells. SOD activity was three-fold higher in stromal endometriotic cells (*p* < 0.01) and 2.25-fold higher in stromal endometrial cells from patients (*p* < 0.05) than in stromal control cells. In epithelial endometrial cells, production of hydrogen peroxide was 7.75-fold higher than in control cells (*p* < 0.0001) and 3.1-fold higher than in epithelial endometrial cells (*p* < 0.001). Catalase activity was 2.7-fold lower in stromal endometriotic cells than in control cells (*p* < 0.01) and 1.8-fold lower than in stromal endometrial cells (*p* < 0.01). Catalase activity was three-fold lower in epithelial endometriotic cells (*p* < 0.01) and 1.5-fold lower in epithelial endometrial cells (*p* < 0.05) compared with control cells. The level of GSH was higher in stromal endometriotic cells than in stromal control cells, and higher in epithelial endometriotic cells compared with epithelial control cells. /This work showed that endometriotic cells from patients with endometriosis have an altered phenotype of ROS production, leading to an increase in the proliferative capabilities of cells.
Ray et al., 2015 [36]	Human study, animal study	Peritoneal fluid was collected from women aged 18–60 years undergoing tubal ligation or laparoscopy for endometriosis (50 women per group). Additionally, eight CD-1 mice and Sprague–Dawley rats were used for the study.	Limulus amebocyte lysate assay, LC-MS/MS, spectrophotometry, agarose gel electrophoresis, enzyme immunoassay, body temperature assay, Hargreaves paw withdrawal pain assay	Prostaglandins, lipoproteins	Increased levels of non-enzymatically derived 8-isoprostanes (*p* = 0.005) were observed in the PF of women with endometriosis compared to controls. Levels of 12,15- or 15-lipoxygenase derived eicosanoids such as 12,15-HETEs (*p* < 0.05) and 5-HETEs, and cyclooxygenase derived eicosanoids such as PGE_2_ and PGD_2_, were higher in endometriosis patients than in control subjects. LC-MS/MS studies confirmed that non-enzymatic oxidation of LDL generates prostaglandin-like molecules. In the animal study, oxidatively modified lipoproteins induced pain-related behavior (significant reductions in withdrawal latencies, indicative of increased pain sensitivity). /This study demonstrated that non-enzymatic oxidatively modified lipoproteins are similar to prostaglandins in their ability to modulate body temperature, induce nociception, and alter the expression of inflammatory and nociceptive genes.
Hevir et al., 2013 [37]	In vitro	Sixty specimens were collected: 31 ovarian endometriomas removed via laparoscopic surgery and 29 control eutopic normal endometrium tissue samples.	Quantitative real-time PCR, Western blotting, and immunohistochemical staining	Genes encoding five estrogen hydroxylating, five OH-estrogen conjugating, and three estrogen quinone detoxifying enzymes (CYPB1, COMT, NQO1, GSTP1, SULTs)	Increased expression of CYP1A1, CYP3A7, and COMT, and higher levels of MB-COMT were seen in endometriosis compared to normal endometrium. Expression of CYP1B1, CYP3A5, SULT1A1, and NQO2 was unchanged, with comparable CYP1B1 protein levels. Expression of SULT1E1, SULT2B1, UGT2B7, NQO1, and GSTP1 was decreased. Three NQO1 isoforms were detected; NQO1c appears to be endometriosis-specific. /The disturbed balance between phase I and II enzymes demonstrated by the study may result in excessive OH-estrogens and ROS formation, resulting in the stimulation of the proliferation of ectopic endometrium.
Chen et al., 2017 [38]	Experimental study	Thirty endometriosis and 30 normal endometrial tissue samples.	Methylation-specific PCR, RT-PCR, Western blot, cell culture	ARID1A, H_2_O_2,_ MDA, GPx	The low level of the ARID1A gene was associated with hypermethylation of its promoter. In H_2_O_2_-stimulated endometrial cells, ARID1A gene expression was decreased. Finally, ROS regulated ARID1A gene expression by changing the methylation level of the ARID1A gene promoter. Finally, both the mRNA and protein levels of DNMT1 were increased in H_2_O_2_-stimulated endometrial cells /In this study, ROS-induced ARID1A gene downregulation was caused by hypermethylation of its promoter. Hypermethylation of the ARID1A gene promoter caused by oxidative stress, leading to low ARID1A gene expression, constitutes a new mechanism of endometriosis.
Chen et al., 2019 [39]	Observational study	Thirty-five childbearing-age women undergoing hysteroscopy for other benign gynecological diseases without endometriosis as a control group, and 78 ectopic endometrial tissues obtained from ovarian endometriotic lesions, and their homologous eutopic endometrium (*n* = 38).	Cell culture, flow cytometry, transmission electron microscopy, Seahorse XF96 extracellular flux analysis, immunohistochemistry, RT-qPCR, Western blotting, cell proliferation assay, cell migration assay	SOD2	SOD2 expression in ectopic endometrium was higher than that in eutopic endometrium (*p* < 0.0001) and controlled endometrium (*p* < 0.0001). SOD2 mRNA expression was significantly elevated in ectopic ESCs compared to eutopic ESCs (*p* < 0.01) and controlled ESCs (*p* < 0.01). SOD2 protein expression was also higher in ectopic ESCs than in eutopic and control ESCs. Mitochondrial membrane potential (MMP) was significantly decreased in si-SOD2-treated cells compared to control cells (*p* < 0.001). /This study identified overexpressed SOD2 coupled with preserved mitochondrial functions but increased mitochondrial superoxide production in endometriosis. The findings indicate that oxidative stress in endometriosis has exhausted the antioxidant mechanisms of the body. Thus, mitochondrial superoxide scavenging can be proposed as a therapeutic option to prevent the development of ovarian endometriosis.
Andrisani et al., 2014 [42]	Observational study	Blood samples were collected from 30 women classified as having endometriosis by histological examination and 27 healthy volunteers serving as the control group.	Immunoassay, carbonic anhydrase assay	GSSP, GSH, carbonic anhydrase	In association with an increase in membrane GSSP and a decrease in cytosolic GSH content in endometriosis patients, carbonic anhydrase monomerization and activity significantly increased (*p* < 0.0001) compared with controls. This oxidation-induced activation of carbonic anhydrase was positively and significantly correlated with the GSH content of RBC (*p* < 0.001) and with the amount of the 30-kDa monomer of carbonic anhydrase (*p* < 0.001). /This study investigated the effects of higher oxidation status on cytosolic carbonic anhydrase in RBC from endometriotic patients. The observation of a close positive correlation between the endometriosis-associated increase in systemic oxidative stress and carbonic anhydrase activity is of great interest as it could potentially be used as the basis for a diagnostic tool to monitor patients’ oxidation status.
Szczepanska et al., 2003 [43]	Retrospective study	Peritoneal fluid samples were collected from 65 women admitted for diagnostic laparoscopy.	Spectrophotometry, ELISA, colorimetry	SOD, GPx, LPO	SOD activity differed significantly between infertile patients with endometriosis and patients with idiopathic infertility (*p* < 0.0001) and fertile controls (*p* < 0.0001). It was lowest in patients with endometriosis and highest in those with idiopathic infertility. Glutathione peroxidase activity was lowest in patients with endometriosis (*p* < 0.000123) and highest in those with idiopathic infertility (*p* < 0.00123). Patients with endometriosis had the lowest total antioxidant status, and those with idiopathic infertility had the highest. Lipid peroxidase levels were highest in women with endometriosis (*p* < 0.039) and lowest in those with idiopathic infertility. /Low antioxidant status and low activity of antioxidant enzymes in the peritoneal fluid of infertile women with endometriosis probably do not influence fertility in these women, but these factors may play a role in the development of the disease.
Ogawa et al., 2022 [44]	In vitro	The cyst walls of ovarian endometrioma were collected from 14 women undergoing surgery for endometriosis.	Cell culture, co-culture experiments, immunocytochemistry, Western blotting, ELISA, cell viability assay, cell proliferation analysis	Macrophage, HO-1, TGF-β1	HO-1 expression was increased in dHTP-1 cells co-cultured with endometriotic cells compared with dHTP-1 monoculture. Co-culturing with dTHP-1 protected endometriotic cells against oxidative injury. Blockade of HO-1 abolished the protective effects of macrophages. /Dynamic cross-talk between endometriotic cells and macrophages may affect the progression of endometriosis through the upregulation of TGF-β1 and HO-1 expression. Macrophage-derived HO-1 protects endometriotic cells from oxidative injury.
Lu et al., 2018 [45]	Randomized controlled study	A total of 280 patients with endometriosis who underwent IVF-ET were enrolled in the study and divided into two groups: a vitamin C treatment group (*n* = 160) and a non-treatment group (*n* = 120). Additionally, 150 patients without endometriosis who also underwent IVF-ET were enrolled as the control group. Plasma and peritoneal fluid samples were collected for analysis.	Phenanthroline colorimetry, thiobarbituric acid chromatometry, spectrophotometry, xanthine oxidase method	SOD, TAC, MDA, ROS	FF levels of VitC, SOD, and TAC were significantly lower in patients with EMs than in controls (*p* < 0.05). FF levels of SOD and TAC in these two groups were significantly higher than serum levels (*p* < 0.05). In contrast, serum MDA level was significantly higher than FF MDA level in patients with EMs and controls (*p* < 0.05). However, the FF MDA level was higher in EM patients than in controls (*p* < 0.05). ROS levels in serum and FF were significantly higher in EM patients than in controls (*p* < 0.05). Treatment with an oral formulation of vitamin C for 2 months improved serum and FF levels of vitamin C in EM patients, but it did not affect oxidative stress markers (SOD, ROS, TAC, MDA). /Treatment with vitamin C oral formulation improved the serum and FF levels of vitamin C but did not affect oxidant stress markers in patients with Ems.
Prieto et al., 2012 [46]	Prospective clinical cohort study	Blood samples were collected from 23 infertile women with endometriosis and 68 controls, including women who were infertile due to tubal factor, male factor, or healthy egg donors.	Spectrophotometry, high-performance liquid chromatography(HPLC), ELISA	Antioxidants consisting of vitamins C, E, SOD, and MDA	In women with endometriosis, lower vitamin C concentrations were found in FF (*p* = 0.03) and a tendency toward reduced SOD activity in plasma (*p* = 0.059) compared with the control group. Vitamin E plasma levels were significantly higher in women with endometriosis (*p* = 0.001), whereas FF levels did not differ between endometriosis patients and controls. Plasma MDA levels were higher in controls than in endometriosis patients, but the difference was not significant. Negative correlations were observed between plasma vitamin C level and number of oocytes retrieved, number of mature oocytes, and number of fertilized oocytes (*p* = 0.036, *p* = 0.0007, and *p* = 0.046, respectively). /These findings suggest a lower antioxidant capacity in infertile women with endometriosis.
Santanam et al., 2013 [47]	Randomized controlled trial	Fifty-nine women (age 19–41 years) were recruited. Group A (*n* = 46) was given a combination of vitamins E and C, and group B (*n* = 13) was given placebo pills.	ELISA	Vitamin C, E	After treatment with antioxidants, 43% of endometriosis patients reported a decrease in chronic pain (*p* = 0.0055), whereas no patient in the placebo group reported a change in chronic pain. Dysmenorrhea and dyspareunia decreased in 37% and 24% of patients treated with antioxidants, respectively, while in the placebo group, dysmenorrhea-associated pain decreased in 4 patients, and there was no change in chronic pain or dyspareunia. /Natural antioxidants such as vitamins E and C at low doses are a highly efficient alternative therapy for relieving chronic pelvic pain in women with endometriosis. The current study also provided in vivo evidence for the global hypothesis that endometriosis is a disease of oxidative stress.
Mier-Cabrera et al., 2008 [48]	Randomized controlled trial	Thirty-four women with endometriosis were randomly assigned to the study (vitamin) group (*n* = 16) or placebo group (*n* = 18).	Ferrous-ion-mediated oxidation of xylenol orange assay, thiobarbituric acid reactive substances assay, and spectrophotometry	Malondialdehyde, lipid hydroperoxide	The plasma concentrations of LOOHs and MDA were significantly decreased in patients treated with antioxidants compared to the placebo group at 4 and 6 months (*p* < 0.05). Significant differences in plasma concentration compared to baseline were observed for MDA at 4 and 6 months (*p* < 0.05), but only at 6 months for LOOHs (*p* < 0.05). In the supplementation group, significant differences were observed between baseline concentrations of the two oxidative stress markers in peritoneal fluid and plasma, and the corresponding plasma concentrations at month 4 (*p* < 0.04). /The findings suggest that vitamin intake influences the peritoneal milieu as well as the peripheral compartment, and that a decrease in peritoneal oxidative stress is to be expected with vitamin intake.
Amini et al., 2021 [49]	Randomized Controlled Trial	Serum and FF samples were collected from 60 reproductive-aged women with pelvic pain who were randomized into two groups: Group A, which received a combination of vitamins C and E (*n* = 30), and Group B, which received placebo pills (*n* = 30).	ELISA	MDA, vitamin C, vitamin E	Women in group B had significantly lower MDA levels than those in group A before the intervention (*p* < 0.01) and after vitamin administration. MDA (*p* = 0.002) and ROS (*p* < 0.001) were significantly reduced compared to placebo, but no change in TAC level was observed. The intergroup comparison showed a more significant reduction in dysmenorrhea, dyspareunia, and chronic pelvic pain VAS score in group A than group B. /The results suggest that supplementation with vitamins C and E effectively reduces systemic oxidative stress indices in women with endometriosis.
Sutrisno et al., 2022 [50]	Animal study	Thirty-two healthy female mice (*Mus musculus*) were divided into a negative control group, an endometriosis group, and treatment groups administered different doses of genistein.	Quantitative colorimetric determination, colorimetric assay	SOD, GPx	SOD levels in the EMT group were significantly lower than in the control group (*p* = 0.006). Genistein significantly increased SOD levels at doses of 0.13 mg (*p* < 0.001) and 0.26 mg (*p* < 0.001) compared to the EMT group. However, in EMT + G3, EMT + G4, EMT + G5, EMT + G6, the levels of SOD were similar to those of the EMT group. The GPx levels in the EMT group were significantly lower than in the control group (*p* = 0.023). Genistein increased GPx levels significantly in all groups administered genistein (*p* = 0.010; *p* = 0.002; *p* < 0.001; *p* < 0.001; *p* < 0.001, respectively). /Genistein has the potential to increase SOD and GPx levels in the peritoneal fluid of a mouse model of endometriosis to prevent oxidative stress.
Rostami et al., 2023 [51]	Randomized controlled trial	Blood serum and FF samples were collected from 50 infertile endometriosis patients presenting for assisted reproductive techniques (ART).	ELISA	MDA, SOD, CAT, TAC, proinflammatory cytokines (IL-1β, IL-6, TNF-α)	Increased serum levels of TAC (*p* = 0.004) and SOD (*p* = 0.010) were observed after AST therapy in the treatment group. In addition, serum MDA (*p* = 0.031) decreased significantly following antioxidant treatment. Furthermore, AST supplementation led to improvements in the number of oocytes retrieved (*p* = 0.043), the number of mature oocytes (*p* = 0.041), and the number of high-quality embryos (*p* = 0.024). /AST could be a promising supplement to combat the oxidative stress and inflammatory reaction associated with endometriosis. AST may also enhance oocyte and embryo quality in endometriosis patients presenting to ART clinics.
Tang et al., 2019 [52]	In vitro	Immortalized human endometrial stromal cells (HESCs)	Cell culture and transfection, qPCR, dual-luciferase reporter assay, cell viability assay, apoptosis assay, caspase-3/7 activity assay, Western blotting, flow cytometry	Hydrogen peroxide, CAT, SOD, GPx	The data suggest that miR-455 is likely negatively correlated with H_2_O_2_-induced apoptosis in HESCs. Flow cytometric analysis indicated that upregulation of miR-455 significantly reduced H_2_O_2_-induced apoptosis in HESCs. miR-455 alleviated the oxidative stress induced by H_2_O_2_ in HESCs, and H_2_O_2_ significantly decreased the activities of SOD, CAT, and GPx. Silencing of FABP4 generated cytoprotective effects against H_2_O_2_ in HESCs. Moreover, overexpression of FABP4 abrogated the miR-455-mediated effects of antioxidative stress in cells. /Given that oxidative stress is implicated in the pathophysiology of endometriosis because it provokes a general inflammatory response in the peritoneal cavity, these findings suggest that miR-455 may be applied as part of a more optimized therapy for endometriosis.
Chaudhury et al., 2013 [53]	Animal study	For the first part of the study, 20 CD-1 strain Swiss Albino mice were selected (10 controls and 10 endometriosis-induced mice). For the second part, an additional 40 mice were included (30 endometriosis-induced mice and 10 controls). Peritoneal tissue and blood samples were collected from these mice for analysis.	Nanoceria synthesis, cell proliferation/cytotoxicity assay, histological examination, immunohistochemistry, thiobarbituric acid assay, luminol-mediated chemiluminescence assay, ELISA	LPO, TAC, MDA	ROS and LPO levels were higher in endometriosis-induced mice compared to controls (*p* < 0.05, 0 < 0.001, respectively), whereas TAC level was higher in controls (*p* < 0.001). Injection of NAC in endometriosis-induced mice did not reduce ROS levels (*p* > 0.05). However, ROS levels were significantly reduced in endometriotic mice treated with nanoceria (*p* < 0.05). A similar trend was observed with regard to LPO and TAC levels; specifically, endometriotic mice treated with nanoceria showed significantly lower LPO and higher TAC levels (*p* < 0.05). Moreover, nanoceria protected against endometriosis-related adverse effects on oocytes, which is critical for successful pregnancy. /Nanoceria showed promising efficacy against endometriosis-related pathogenesis.
Wang et al., 2024 [54]	In vitro, in vivo	Twenty patients were diagnosed with endometriosis, and 10 controls were without endometriosis. Endometriotic stromal cells were isolated from endometrial samples, while menstrual blood endometrial cells (MESCs) were isolated.	Inflammatory factor sequencing, immunohistochemical staining, qRT-PCR, Western blotting, Calcein-AM/PI fluorescence assay, mitochondrial membrane potential assay, glycolytic rate assay, co-immunoprecipitation assay	Osteopontin (OPN)	OPN was significantly upregulated in endometriosis, suggesting that it may play a role in disease progression (*p* < 0.0001). In vitro assays demonstrated significant upregulation of OPN in EESCs (*p* < 0.01), and knockdown of OPN effectively inhibited necroptosis and the release of inflammatory factors by inhibiting mitochondrial stress and ROS release. In vivo, targeting of OPN can inhibit the growth of endometriotic lesions. Clinically, OPN was significantly upregulated in the menstrual blood of patients with endometriosis. /This study unveils the critical role of OPN in regulating the RhoA/ROS signaling pathway, thereby controlling necroptosis and the release of inflammatory factors. OPN knockdown effectively impeded the development of endometriosis, providing a promising treatment approach in which inflammation is targeted through OPN regulation. Additionally, measuring OPN levels in menstrual blood may be a non-invasive and specific method for detecting endometriosis.
Seidita et al., 2023 [55]	In vitro	Immortalized human endometrial stromal cells (HESC).	Cell culture, Western blotting, cell transfection, qRT-PCR, confocal microscopy, flow cytometry	S1P	Treatment with 100 nM S1P for 10 min potently increased ROS formation in HESCs (*p* < 0.001). S1P stimulation increased ROS production, and S1P activated ERK5 through S1P_1_ and S1P_3_ receptor engagement and downstream activation of SFK and MEK5. Activation of ERK5 by the expression of a constitutively active form of MEK5 significantly increased intracellular ROS, confirming the role of MEK5-dependent phosphorylation of ERK5 in the modulation of ROS levels in HESCs. /The findings indicate that S1P signaling, via ERK5 activation, supports a proinflammatory response in the endometrium and establish a rationale for exploiting innovative therapeutic targets for endometriosis.
Lu et al., 2020 [56]	Animal study	Fifty Wistar rats were randomly divided into four groups: an endometriosis group, an endometriosis group treated with normal saline, an endometriosis group treated with N-acetyl-L-cysteine (NAC), and an endometriosis group treated with catalase (CAT). Endometriotic endometrial tissue was collected from these groups for analysis.	Immunofluorescence, Western blotting, ELISA, HE staining	NAC, CAT	LC3 fluorescence levels were significantly lower in the EMs group of rats compared with controls (*p* < 0.05). Western blot analysis revealed a downregulation of Beclin-1 protein in both the NAC and CAT groups compared with controls (*p* < 0.05), while ELISA revealed significantly lower ROS levels in the NAC and CAT groups (*p* < 0.05). /The study demonstrated that hypoxia induced autophagy in EMs cells and resulted in ROS generation, and that these biological changes could be reversed by the antioxidants CAT and NAC. The results may partly explain the mechanism by which the levels of autophagy markers are reduced in response to ROS inhibitors.
Cabrera et al., 2009 [57]	Comparative cross-sectional study	Blood samples were collected from women with endometriosis (WEN, *n* = 83) and without endometriosis (WWE, *n* = 80), who were interviewed for the study. Among the WEN group, participants were further divided into a control group (*n* = 35) and a high antioxidant diet group (*n* = 37).	Xylenol orange assay, thiobarbituric acid assay, electrochemical detection, colorimetric assay	Vitamin A, C, E, zinc, copper, MDA, GPx, SOD	Comparison of antioxidant intake between WWE and WEN showed a lower intake of vitamins A, C, E, zinc, and copper by WEN (*p* < 0.05). Increases in vitamin concentrations (serum retinol, alpha-tocopherol, leukocyte and plasma ascorbate) and antioxidant enzyme activity (SOD, GPx), as well as decreases in oxidative stress markers (MDA, lipid hydroperoxides) were observed in the HAD group after 2 months of intervention. These phenomena were not observed in the control group. /Application of a HAD in women with endometriosis increased the peripheral enzymatic SOD and GPx activity after 3 months of intervention in comparison to the control diet group. Additionally, application of a HAD in women with endometriosis decreased the peripheral concentrations of MDA and LPOs after 3 months of intervention in comparison to the control diet group.

Abbreviations: CAT, catalase; ELISA, Enzyme-Linked Immunosorbent Assay; ERK, Extracellular Signal-Regulated Kinase; FF, follicular fluid; FOX, FORD, Free Oxidant Radical Defense; FORT, Free Oxygen Radicals Test; Ferric Xylenol Orange Assay; GSH, glutathione; GSSG, oxidized glutathione; HO-1, Heme Oxygenase-1; IHC, Immunohistochemical Staining; LC-MS/MS, liquid chromatography-tandem mass spectrometry; LC3, Microtubule-associated Protein 1A/1B-light Chain 3; MDA, malondialdehyde; LOX, lipoxygenase; MDA, Malondialdehyde; MEK5, Mitogen-activated Protein Kinase Kinase 5; MESCs, Menstrual Blood Endometrial Cells; MMP-3, Matrix Metalloproteinase-3; NAC, N-acetyl-L-cysteine; NOX2, NADPH Oxidase 2; NRROS, Negative Regulator of Reactive Oxygen Species; Nrf2, Nuclear Factor Erythroid 2-Related Factor 2; NSAIDs, Non-Steroidal Anti-Inflammatory Drugs; O_2_^−^, Superoxide Anion; OCl^−^, Hypochlorite; OPN, Osteopontin; p16^INK4a^, Cyclin-dependent Kinase Inhibitor 2A; PGD2, Prostaglandin D2; PGE2, Prostaglandin E2; PGC-1α, Peroxisome Proliferator-Activated Receptor Gamma Coactivator 1 Alpha; PKM2, Pyruvate Kinase M2; PMN, Polymorphonuclear Leukocytes; qRT-PCR, Quantitative Reverse Transcription Polymerase Chain Reaction; RANTES, Regulated on Activation, Normal T cell Expressed and Secreted (CCL5); ROS, Reactive Oxygen Species; SOD, Superoxide Dismutase; TAC, Total Antioxidant Capacity; TGF-β1, Transforming Growth Factor Beta 1; TLR1, Toll-Like Receptor 1; TLR2, Toll-Like Receptor 2; TLR4, Toll-Like Receptor 4; TNF, Tumor Necrosis Factor; TNF-α, Tumor Necrosis Factor Alpha; Ucp2, Uncoupling Protein 2; VEGF, Vascular Endothelial Growth Factor; Vitamin C/E, Vitamin C and Vitamin E.

**Table 5:** Research studies suggesting that ROS are not related to the pathogenesis of endometriosis.

Author/ Year/ Reference	Study Design	Species and/or Sample	Detection Method	Target Substances	Results/Conclusions
Arumugam et al., 1995 [58]	In vitro	Twelve peritoneal fluid samples from patients with moderate to severe endometriosis, 15 samples from patients with minimal to mild endometriosis, and 13 samples from patients with a normal pelvis	Thiobarbituric acid assay	LPO, MDA	LPO levels were not affected by the presence or severity of endometriosis. The mean values for the MDA were: 1.09 ± 0.14 for moderate to severe endometriosis, 0.95 ± 0.09 for minimal to mild endometriosis, and 1.07 ± 0.15 for normal pelvis. /The TBA assay results show LPO levels in the pelvic cavity are independent of the presence or absence of endometriosis. As such, LPOs in the peritoneal fluid do not appear to play a significant role in the causal relationship between endometriosis and infertility.
Amaral et al., 2004 [59]	Prospective study	Peritoneal fluid samples from 21 infertile women with endometriosis and 21 patients undergoing tubal ligation	Thiobarbituric acid assay	MDA, LPO	A significant increase (*p* < 0.01) in MDA levels was observed in the peritoneal fluid after the addition of copper in both groups (MDA and MDA/Cu^2+^ = 0.07 and 0.34 nmol/mL, and 0.04 and 0.21 nmol/mL in patients with pelvic endometriosis and controls, respectively). /These data suggest that increased ROS may not be one of the factors responsible for compromised fertility in patients with endometriosis.

Abbreviations: MDA, malondialdehyde; ROS, reactive oxygen species; TBA, thiobarbituric acid assay.
